# The Effects of Individualized Information and Emotional Support Education on Midwifery Students’ Anxiety during the COVID-19 Pandemic

**DOI:** 10.3390/medicina58101376

**Published:** 2022-10-01

**Authors:** Zehra Baykal Akmeşe, Birsen Karaca Saydam, Güzin Kardeş

**Affiliations:** Department of Midwifery, Karşıyaka Suat Cemile Balcıoğlu Yerleşkesi, Ege University Faculty of Health Science, Izmir 35575, Turkey

**Keywords:** anxiety, COVID-19, emotional support, individualized information, midwifery students, pandemic

## Abstract

*Background and Objectives*: Midwifery students were not able to participate in internship programs and related practices during the COVID-19 pandemic. This kept them from meeting graduation requirements because they could not do the one-on-one observations for clinical case management. In this study, we aim to determine the effects of Individualized Information and Emotional Support Education (IESE) on the midwifery students’ anxiety levels during the COVID-19 pandemic. *Materials and Methods*: This is an experimental study with two stages. In the first stage, the researchers determined the anxiety levels of 268 students. In the second stage, 76 students with high levels of anxiety were provided with IESE. The IESE was conducted in interviews on online platforms and took a minimum of 60 min. The students’ anxiety levels were measured again one week after the IESE. *Results*: Statistically significant differences were found between the students’ State Anxiety Scale scores before and after the IESE (t = 8.756, *p* = 0.000). Before the IESE, 65.8% of the students had high anxiety levels about COVID-19-related disease or death, and the possibility of losing loved ones. After the education, this rate fell by 17.1% to 48.7%, and this difference was significant (χ^2^ = 5.077, *p* = 0.024). *Conclusions*: The IESE positively affected the students’ anxiety levels. Even just showing interest can make people feel cared for and valued, and people are sensitive to their needs. After this study, 30 students with high anxiety levels were sent for consultation with an expert and have been followed up by researchers.

## 1. Introduction

The novel coronavirus disease (COVID-19) first appeared in Wuhan, China, in 2019, as cases of pneumonia of unknown origin, and was declared a pandemic in 2020 by the World Health Organization [1,2]. The COVID-19 pandemic has quickly become a serious threat to global health and adversely affected various sectors, such as transportation, education, business and health [3,4]. In order to prevent the COVID-19 epidemic, social isolation rules were applied for the first time in countries and all areas were crowded groups gathered were closed [5,6]. At the same time, many other measures were implemented, such as the use of masks, curfew, quarantine practices, the closing of cafes and restaurants, and the interrupting of face-to-face education at universities and other educational institutions [7,8].

The suspension of face-to-face education during the pandemic has caused damage to education and training processes. It has been reported that 90% of the 1.5 billion young people enrolled in any school all over the world could not continue their education due to the pandemic [9]. The United Nations Educational, Scientific and Cultural Organization (UNESCO) has declared that education should be continued through distance learning, and countries will be supported in reducing the negative effects of the suspension of face-to-face education [5]. The United Nations International Children’s Emergency Fund (UNICEF) has recommended that access to distance education methods should be provided for all students without internet access, including students with disabilities, to ensure that education is not interrupted [10]. In the case of Turkey, face-to-face education was suspended for some time at all education levels, and distance education opportunities were developed and offered to students at every education level [11,12]. The Council of Higher Education (CoHE) issued the decision (Information Note on COVID-19) declaring that face-to-face education would be suspended for three weeks in all higher education institutions starting on 16 March 2020. Then, the CoHe announced the suspension of face-to-face education for the spring term of the 2019–2020 academic year [13]. Especially with the suspension of face-to-face education in universities, and with the transition to distance education, the number of cases and the burden on the health system have been reduced [6,14]. However, the pandemic has also been challenging for universities as it has led to the disappearance of the traditional education system [15,16]. The effort to adapt both to the pandemic and to the distance education processes has caused students to experience some mental problems [17,18,19]. Social isolation, physical and social contact restrictions, the fear of illness and the loss of close relatives increased the risk of students developing anxiety and depression [20,21]. It was stated that 220 million students in China stayed at home for a long time due to the pandemic, and 18.6% of them showed symptoms of anxiety [21]. Studies have shown that the risk of anxiety, stress and depression in young people is much higher than in adults during the pandemic [22,23,24].

It was indicated that education could be conducted online to prevent aggrievement and the loss of learning for students in departments with applied courses, such as midwifery [13]. However, in line with the European Union Midwifery Directives, in order to receive the title of ‘midwife’, midwifery students must complete targeted practices, such as 100 prenatal examinations, 40 births, 100 postpartum and newborn examinations, and the follow-up of 40 risky pregnancies during the four-year undergraduate program. Midwifery students are required to actively participate in field and clinical internships in order to complete the practices. However, distance education processes for the midwifery program during the COVID-19 pandemic may cause students not to be able to actively participate in internships, to observe and practice, and to experience anxiety due to the possibility of extending the term, or not completing the program [25,26,27,28]. It has been determined that midwifery students in the north of Turkey experienced feelings such as loneliness and uncertainty, developed various coping mechanisms on their own, and experienced inadequacy and anxiety in their professional skills [29,30]. It was stated that midwifery students in the central Anatolian region of Turkey experienced moderate fear due to COVID-19, and it was suggested that structured, individual information and support programs should be organized [31]. Studies have shown that providing remote support to midwifery students can help to detect and eliminate problems that may cause serious mental problems at an early stage [32,33,34,35]. In this study, it was thought that it would cognitively relax students with high levels of anxiety by informing them about the solution of problems related to the COVID-19 pandemic, and how to carry out the process of staying at home/adaptation and distance education. This was accompanied by a guide prepared by the Ministry of Health Science Committee. It was envisaged that increasing bodily awareness with breathing and relaxation exercises would prevent students from experiencing loneliness and uncertainty, thus reducing their anxiety. Therefore, the aim of this study is to reveal the effect of the 60 min ‘Information and Emotional Support Training’ created by researchers for midwifery students with anxiety.

## 2. Materials and Methods

### 2.1. Ethics

All procedures performed in studies involving human participants were in accordance with the ethical standards of the institutional and/or national research committee, and with the 1964 Helsinki Declaration and its later amendments, or comparable ethical standards. All procedures were approved by the Ministry of Health’s Directorate General for Health Services Scientific Research Platform (3 June 2020), the University Deanship of the Faculty of Medicine Medical Research Ethics Committee (10 June 2020), the University Faculty of Health Sciences (28 May 2020) and the Head of the Department of Midwifery (28 May 2020).

### 2.2. Study Design

This quasi-experimental study aimed to determine the effects of IESE on midwifery students’ anxiety levels during the COVID-19 pandemic. The population of the study included all the students in the Department of Midwifery in one of the largest university’s in the west of Turkey, during the 2019–2020 academic year (401). Its sample consisted of 268 students who agreed to participate. Of the target population, 68.8% were reached.

### 2.3. Data Collection

In the first stage of data collection, the researchers contacted 268 students in the University Faculty of Health Sciences Department of Midwifery, during the 2019–2020 academic year. First, class representatives gave the students a brief explanation about the study. Then, from 11 May to 18 May 2020, links for data collection forms were sent to the students social media accounts (Instagram, WhatsApp and Facebook) and e-mail addresses, using Google Forms (https://forms.gle/FuVAwGYNCZfxSakEA accessed on 11–18 May 2020). The students who agreed to participate in the study were asked to fill out the forms carefully within a week. Then, the data were analyzed. Eighty students who scored 50 or higher were considered to have high anxiety levels.

**Figure 1:**
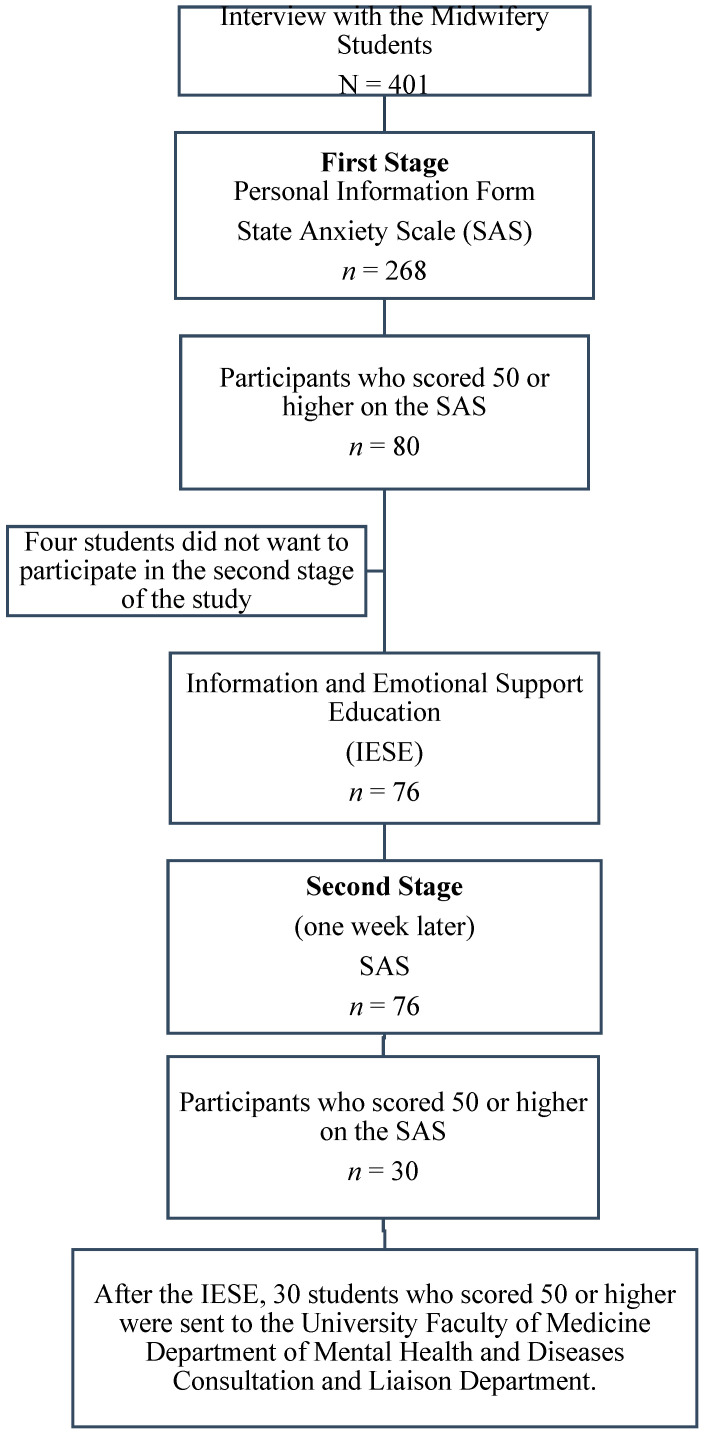
Flow chart of data collection.

The second stage of the study was conducted with 76 students who had high anxiety levels and who agreed to proceed. Four students left the study at this point. Dates and video call platforms suitable for the students were determined through individual interviews. The students were provided with at least 60 min of Individualized Information and Emotional Support Education (IESE) by the researchers between 1 and 12 June 2020. One week after the IESE was provided to each student, between 20 and 27 June 2020, the State Anxiety Scale (SAS) was administered again by sending links to the students’ social media accounts (Instagram, WhatsApp and Facebook) and e-mail addresses using Google Forms (https://forms.gle/FuVAwGYNCZfxSakEA accessed on 20–27 June 2020). After the second measurement, 30 students who scored 50 or higher on the SAS were sent to the University Faculty of Medicine Department of Mental Health and Diseases Consultation and Liaison Department (Figure 1).

*Administration of the IESE:* The students who scored 50 or higher on the SAS were contacted by e-mail. After they were informed about the objective of the study and its requirements, their verbal consent was obtained once again. Then, suitable dates and video call platforms for the IESE were determined to allow the researchers and students to communicate easily. The individualized IESE was conducted in one-to-one interviews by video call (Skype, Teams, Zoom and Google Meet) in a quiet environment for approximately 60 min.

### 2.4. Data Collection Tools

*The Personal Information Form:* This form has 25 questions about the student’s age, their parents’ employment, social security, family health and income, the number of siblings, a history of mental disorders and situations that can cause anxiety.

*State Anxiety Scale (SAS):* This study used the 20-item State Anxiety Scale (SAS), which is a part of the 40-item State-Trait Anxiety Inventory, developed by Charles D. Spielberger, Richard L. Gorsuch and Robert E. Lushene in 1964 [36,37,38]. The SAS has been translated into more than 30 languages and used by many researchers. Öner and Le Compte did the validity and reliability study of the Turkish version and found that its Cronbach’s alpha coefficient ranged from 0.94 to 0.96. The SAS measures how individuals feel at a certain time and in specific conditions. Scores are based on ratings for each item: 1 (not at all); 2 (somewhat); 3 (moderately so); and 4 (very much so). The scale has 10 reverse-scored items: 1, 2, 5, 8, 10, 11, 15, 16, 19 and 20. The lowest possible score is 20, and the highest is 80 [39]. Higher scores indicate higher levels of anxiety. Some of the clinical studies have suggested that its cut-off point should be 39–40 [40,41]. Others have suggested that it should be 54–55, or higher [42]. The cut-off point was 50 or higher in this study [43,44,45]. The Cronbach’s alpha coefficient was 0.92 for the first administration of the SAS.

### 2.5. Data Analysis

Data analysis was carried out using the Statistical Package for the Social Sciences (SPSS) 20.0 (IBM Inc., Armonk, NY, USA). The Kolmogorov-Smirnov test was used to determine whether the data were normally distributed. The data are shown as numbers, percentages, means and standard deviations. The *t*-test, ANOVA and the chi-squares test were used for the independent groups. The threshold for statistical significance was *p* < 0.05.

## 3. Results

### 3.1. Descriptive Statistics

The students’ mean age was 20.75 ± 1.92 (min = 18.0-max = 29.0) years. Of them, 76.1% were in the 20–24 age group, 79.9% had completed Anatolian or science high schools, and 87.7% had social security. Of the students, 36.9% were living in districts, and 81.7% had nuclear families. Of the students’ parents, 64.9% of their fathers and 25.4% of their mothers were employed. Of the students, 39.2% had two or three siblings, and 62.7% had equal income and expenses. In response to a multiple-choice question, 60.8% of the students described their general emotional state as unhappy, anxious or unemotional, and 39.2% said that they were happy.

Of the students, 24.6% were freshmen, 32.8% were sophomores, 25.7% were juniors and 16.8% were seniors. Of the students, 47.8% were on scholarships and 41.8% had national scholarships. Of the students, 54.1% had acquaintances who were health care professionals, and 56.7% had chosen the midwifery department of their own accord. Of the students, 76.1% were happy with the midwifery department, and 72.4% said that their academic achievement level was moderate. Of the students, 14.6% had family members with mental disorders.

*The IESE’s Effects on the Midwifery Students’ Anxiety Levels.*

*Before the IESE:* The students’ (n = 268) mean SAS score was 44.4 ± 10.4 (min = 20.0-max = 76.0) Of the students, 70.1% (188) had low levels of anxiety ($\bar{X}$ = 39.1 ± 6.64 min = 20.0-max = 49.0), and 29.9% (80) had high levels of anxiety ($\bar{X}$ = 57.0 ± 6.27 min = 50.0-max = 76.0) (Table 1).

*After the IESE:* Four students with high levels of state anxiety did not agree to participate in the second stage of the study. The mean SAS score of the students (n = 76) was 46.2 ± 12.0 (min = 23, max = 74). Statistically significant differences were found between the students’ mean SAS scores before and after the IESE (t = 8.756, *p* = 0.000) (Table 2). Before the IESE, the most common cause of anxiety (65.8%) was worry about COVID-19-related disease or death and the possibility of losing loved ones. After the education, this rate fell by 17.1% to 48.7%, and this difference was significant (χ^2^ = 5.077, *p* = 0.024).

### 3.2. Comparison of the Midwifery Students’ Mean SAS Scores and Some Sociodemographic Variables

Statistically significant correlations were found between the students’ mean SAS scores before the IESE, by high school (*p* = 0.001) (Anatolian or science), perception of income and expenses (*p* = 0.0013) (income equal to expenses), history of mental disorders in the family (*p* = 0.000) (those who did not have such a history) and emotional state (*p* = 0.000) (those who were unhappy, anxious or unemotional). There was a statistically significant correlation between the students’ mean SAS score after the IESE and their mothers’ employment status (*p* = 0.006) (those who were not employed) (Table 3).

The students’ mean SAS scores before and after the IESE were compared by age group (before *p* = 0.511, after *p* = 0.312), longest place of residence (before *p* = 0.884, after *p* = 0.774), family type (*p* = 0.385 and *p* = 0.725), number of siblings (before *p* = 0.385, after *p* = 0.962), father’s employment status (before *p* = 0.712, after *p* = 0.330) and social security status (before *p* = 0.698, after *p* = 0.790). These variables did not cause statistically significant differences in their mean SAS scores (Table 3).

## 4. Discussion

This study revealed that the effect of education by giving individualized ‘Information and Emotional Support Education’ (IESE) to midwifery students with high anxiety levels during COVID-19. Students of the midwifery department of one of the largest universities in Turkey were included in the research. However, the entire target population could not be reached due to the suspension of the education that began with the first case in Turkey [11,13]. In addition to primary, secondary and higher education schools, during the pandemic the preparation process required for the acquisition of the tools and equipment necessary for reaching distance education in many families could not be experienced, and it took time to eliminate the deficiencies due to curfew restrictions. In studies examining the problems experienced by students during the distance education process during the COVID-19 pandemic period, it was determined that students had difficulties in distance education due to reasons such as low socio-economic level, living in the countryside, and the lack of internet and technological tools at home [17,27,32,46]. In this study, 70% of the participants were reached. In addition to the reasons stated in previous studies, the fact that nearly half of the students have two or three siblings, and the majority of them live in a small town, suggests that they may experience a lack of internet and related equipment.

Cao et al. (2020) reported in their study that students stated the possibility of their relatives and themselves being infected as the most important cause of anxiety in the pandemic, and that they experienced stress and had to postpone their academic studies because of this [47]. Research has revealed that the uncertainty caused by the pandemic and the anxiety about how to protect oneself made it difficult for students to continue distance education [9,21,25,46]. In our study, the reasons for anxiety in students with high anxiety levels were determined as ‘illness/death due to COVID-19 and loss of relatives’, supporting the literature. It has also been reported by research conducted with medical students in the USA, and with university students in Saudi Arabia during the pandemic, that past and present mental health conditions are an important predictor of students’ mental health during the pandemic [48,49]. In this study, the anxiety levels of the students who had a family history of mental illness and described their emotional state as unhappy/anxious/emotional were found to be significantly higher. It can be thought that the family history of mental illness and a negative emotional state may cause an increase in the anxiety levels of midwifery students, who are thought to be weaker in terms of mental health, due to the effect of the pandemic.

The COVID-19 pandemic has dramatically increased the prevalence of anxiety among college students [49,50,51,52]. In the report published by the Organization for Economic Co-operation and Development (OECD) in 2021, it was reported that intervention support programs prepared to prevent increasing mental problems in young individuals during the COVID-19 pandemic, were effective [51,53]. Similarly, it has been emphasized in studies that cognitive and emotional programs should be developed and implemented for particularly vulnerable individuals [50,51,54,55,56]. Research also reveals that interventions to protect and improve mental health have become mandatory, both during and after the pandemic [49,57,58,59]. An eight-week online intervention program (ETUCARE), developed in France to improve the mental health of university students, was found to increase students’ psychological well-being and reduce anxiety [34]. Another study with university students in Australia examined the effect of a five-week program on the outcome of positive mental health (i.e., well-being, life satisfaction, resilience) and the indicators of psychological distress (i.e., depression, anxiety, stress). In the study, it was determined that the program applied for five weeks significantly improved all measured mental health outcomes and showed low and medium effect sizes on individual outcomes [35]. In a study evaluating the effect of a psychosocial adjustment program on the anxiety and mental well-being of 91 midwifery students during the COVID-19 pandemic in the northeast of Turkey, it was found that the program reduced their state and trait anxiety, and increased their psychological well-being [33]. In this study, video calls (Skype, Teams, Zoom, Google-meet) lasting 60 min were conducted with students whose state anxiety scale score was 50 and above, and structured, individualized information and emotional support training was applied in the interview. It was determined that the anxiety of midwifery students decreased after the education; while the research result is similar to the literature, it also provides evidence of the significant impact of remote information and emotional support during COVID-19.

On the other hand, the fact that this study was conducted with students studying in a single faculty, and who could only be online during the time period when the data were collected and with access to the internet, can be considered as a limitation in terms of the representation of the population of the sample. However, the place where the research was conducted is İzmir, the third largest city of the country, and located in the west of Turkey. The midwifery department of the Faculty of Health Sciences of the university is one of the most preferred universities by students from every region of Turkey. Based on the assumption that the students represent every region of Turkey, it was felt to be important to include all the students of the midwifery department within the Faculty of Health Sciences in the scope of the research. This study may also have a number of non-measured outcomes. One of these is that this experimental study was designed using a pragmatic approach that encouraged the students to get professional support when they felt the considerable effects of the pandemic. The IESE allowed the students to express their anxiety, to increase their physical awareness, and to learn that anxiety can be controlled by stimulating muscle groups in different parts of the body. This awareness can help students to develop coping strategies for future life crises. The IESE can serve as a model for lecturers counseling sessions.

## 5. Conclusions

The individualized IESE provided to midwifery students during the COVID-19 pandemic effectively reduced their state anxiety scale levels. Stressful situations can put stable lives at risk. In this study, an imbalance of income and expenses, and not having family members with mental disorders, were the main determinants of the students’ state anxiety scale levels. Anxiety due to the COVID-19 pandemic can be addressed through care and education.

During the writing of the study, the researchers found that four of the 30 students with high levels of anxiety after the IESE started receiving appropriate treatment. The other 26 students were continuing interviews with a psychologist. The researchers are still in contact with the students.

## Figures and Tables

**Table 1:** The distribution of the students’ mean state anxiety scale scores.

Mean State Anxiety Scale Scores	Before the IESE *				After the IESE *			
	*N*	%	Lowest-Highest	Mean/SD	*N*	%	Lowest-Highest	Mean/SD
Low level of anxiety (49 or lower)	188	70.1	20.0–49.0	39.1 ± 6.64	46	60.5	23.0–49.0	38.3 ± 7.30
High level of anxiety (50 or higher)	80	29.9	50.0–76.0	57.0 ± 6.27	30	39.5	50.0–74.0	58.3 ± 6.35
Total score	268	100	20.0–76.0	44.4 ± 10.4	76	100	23.0–74.0	46.2 ± 12.0

IESE *: Individualized Information and Emotional Support Education.

**Table 2:** Comparison of the participants’ state anxiety scale scores before and after the IESE.

Mean State Anxiety Scale Scores	Before the IESE *		After the IESE *		t	*p*
	*N*	Mean/SD	*N*	Mean/SD		
High level of anxiety (50 or higher)	76	57.1 ± 6.32	76	46.2 ± 12.0	8.756	0.000

IESE *: Individualized Information and Emotional Support Education.

**Table 3:** Comparison of the participants’ mean SAS scores and their sociodemographic variables before and after the IESE.

Variables		SAS Score before IESE				SAS Score after IESE			
		*N*	Mean ± SD	F/t	*p*	*N*	Mean ± SD	F/t	*p*
Age group	15–19	59	43.32 ± 9.67	0.674 *	0.511	15	44.20 ± 8.92	1.182 *	0.312
	20–24	204	44.88 ± 10.45			59	46.32 ± 12.65		
	25–29	5	41.8 ± 20.40			2	58.00 ± 8.48		
High school	Anatolian/science high school	214	45.67 ± 10.48	7.194 *	0.001	67	45.92 ± 12.03	2.422 *	0.096
	Medical vocational high school	30	39.50 ± 8.10			4	39.00 ± 11.57		
	Regular high school	24	40.08 ± 10.58			5	55.8 ± 6.72		
Longest place of residence	Town/Village	48	44.00 ± 9.03	0.884 *	0.450	13	45.15 ± 11.72	0.371 *	0.774
	District	99	44.71 ± 10.74			30	46.30 ± 12.49		
	Province	47	46.38 ± 12.04			17	48.58 ± 10.71		
	Metropolis	74	43.28 ± 9.99			16	44.37 ± 13.26		
Family type	Extended family	34	46.79 ± 9.95	0.957 *	0.385	12	48.33 ± 11.95	0.322 *	0.725
	Nuclear family	219	44.11 ± 10.57			58	46.03 ± 12.16		
	Fragmented family	15	44.60 ± 10.58			6	43.66 ± 11.91		
Number of siblings	None	19	45.10 ± 12.43	0.957 *	0.385	4	48.25 ± 22.00	0.096 *	0.962
	1	102	44.14 ± 10.37			27	46.33 ± 11.97		
	2–3	105	44.20 ± 10.81			32	45.50 ± 12.14		
	4 or more	42	45.71 ± 9.25			13	47.07 ± 9.22		
Perception of income and expenses	Equal income and expenses	71	47.14 ± 11.06	4.384 *	0.013	27	49.00 ± 11.03	1.208 *	0.305
	Less income than expenses	168	44.00 ± 10.10			45	44.86 ± 11.88		
	More income than expenses	29	40.75 ± 10.10			4	42.50 ± 19.07		
Mother’s employment status	Employed	68	46.19 ± 11.32	1.556 **	0.121	20	52.45 ± 11.57	2.831 **	0.006
	Unemployed	200	43.90 ± 10.16			56	43.98 ± 11.45		
Father’s employment status	Employed	174	44.31 ± 10.73	−0.370 **	0.712	44	47.36 ± 12.84	0.981 **	0.330
	Unemployed	94	44.80 ± 10.09			32	44.62 ± 10.74		
Social security	Yes	235	44.39 ± 10.79	−0.398 **	0.698	67	46.07 ± 12.44	−0.267 **	0.790
	No	33	45.15 ± 8.17			9	47.22 ± 8.51		
History of mental disorders in the family	Yes	39	51.07 ± 11.10	4.385 **	0.000	21	45.85 ± 13.98	−0.157	0.875
	No	229	43.36 ± 9.98			55	46.34 ± 11.30		
Emotional state	Unhappy/Anxious/Unemotional	163	48.19 ± 10.76	8.005 **	0.000	-	-	-	-
	Happy	105	38.73 ± 6.88			-	-		
TOTAL		268	44.4 ± 10.4			76	46.2 ± 12.0		

* Variance (ANOVA) analysis; ** independent groups *t*-test.

## Data Availability

Not applicable.
